# Correction: Varela et al. Potential Benefits of Lycopene Consumption: Rationale for Using It as an Adjuvant Treatment for Malaria Patients and in Several Diseases. *Nutrients* 2022, *14,* 5303

**DOI:** 10.3390/nu16203503

**Published:** 2024-10-16

**Authors:** Everton Luiz Pompeu Varela, Antônio Rafael Quadros Gomes, Aline da Silva Barbosa dos Santos, Eliete Pereira de Carvalho, Valdicley Vieira Vale, Sandro Percário

**Affiliations:** 1 Oxidative Stress Research Laboratory, Institute of Biological Sciences, Federal University of Pará, Belém 66075-110, Brazil; rafaelquadros13@hotmail.com (A.R.Q.G.); aline.barbosa@ics.ufpa.br (A.d.S.B.d.S.); elicar28@uol.com.br (E.P.d.C.); percario@ufpa.br (S.P.); 2 Post-Graduate Program in Biodiversity and Biotechnology of the BIONORTE Network, Federal University of Pará, Belém 66075-110, Brazil; 3 Post-Graduate Program in Pharmaceutical Innovation, Federal University of Pará, Belém 66075-110, Brazil; valdicleyvale@gmail.com

The journal’s Editorial Office and Editorial Board are jointly issuing a resolution and removal of the *Journal Notice* linked to this article [1], as well as an update to the original publication. Following concerns raised about the integrity of the peer review, the Editorial Office has conducted a post-publication peer review of this article. This process included the recruitment of two new independent reviewers and was supervised by one of the Editorial Board members and the Editor-in-Chief to ensure full compliance with MDPI’s Editorial Process (https://www.mdpi.com/editorial_process, accessed on 4 October 2024).

As a result of this process, the Editor-in-Chief and the authors have agreed to update the following aspects of this publication:

Reviewer report 1 was removed from the peer review record, and two new reports were uploaded to the peer review record (https://www.mdpi.com/2072-6643/14/24/5303/review_report).

## 1. Update to the Academic Editor

One additional Editorial Board Member Jean Christopher Chamcheu has been involved in the process of the post-publication review, and the original Academic Editor has been updated and has agreed with the final decision.

## 2. Text Correction

The last sentence in Section 4.2 was changed to the following:

During absorption, lycopene taken up by the enterocyte can also be cleaved by β-carotene 9′,10′-oxygenase (BCO2) to form apo-lycopenoids, including apo-lycopenal, -lycopenol, and -lycopenoic acid [181–183].

In Section 4.3, the term “apolycopenols” was replaced with “apo-lycopenoids”.

In Section 6, the phrase “vitamins A and C, retinol, β-carotene, α-carotene, β-cryptoxanthin, lutein, and lycopene” was changed to “vitamins A (retinol) and C, β-carotene, α-carotene, β-cryptoxanthin, lutein, and lycopene”.

## 3. Citation Removal

The reference [268] Zeba, A.N.; Sorgho, H.; Rouamba, N.; Zongo, I.; Rouamba, J.; Guiguemdé, R.T.; Hamer, D.H.; Mokhtar, N.; Ouedraogo, J.B. Major Reduction of Malaria Morbidity with Combined Vitamin A and Zinc Supplementation in Young Children in Burkina Faso: A Randomized Double Blind Trial. *Nutr. J.* 2008, *7*, 1–7. https://doi.org/10.1186/1475-2891-7-7 has been removed. With this correction, the order of some references has been adjusted accordingly.

With this update, the Academic Editor is satisfied that the Editorial Process relating to this article has been completed as per MDPI’s Editorial Process policy. The Editorial Office would like to thank the authors for their collaboration during this process.

## References

[1] Varela E.L.P., Gomes A.R.Q., da Silva Barbosa dos Santos A., de Carvalho E.P., Vale V.V., Percário S. (2022). Potential Benefits of Lycopene Consumption: Rationale for Using It as an Adjuvant Treatment for Malaria Patients and in Several Diseases. Nutrients.

